# Cytokine Response Signatures in Disease Progression and Development of Severe Clinical Outcomes for Leptospirosis

**DOI:** 10.1371/journal.pntd.0002457

**Published:** 2013-09-19

**Authors:** Eliana A. G. Reis, José E. Hagan, Guilherme S. Ribeiro, Andrea Teixeira-Carvalho, Olindo A. Martins-Filho, Ruth R. Montgomery, Albert C. Shaw, Albert I. Ko, Mitermayer G. Reis

**Affiliations:** 1 Laboratory of Pathology and Molecular Biology, Oswaldo Cruz Foundation, Salvador, Bahia, Brazil; 2 Department of Epidemiology of Microbial Diseases, Yale School of Public Health, New Haven, Connecticut, United States of America; 3 Institute of Collective Health, Federal University of Bahia, Salvador, Bahia, Brazil; 4 René Rachou Institute, Oswaldo Cruz Foundation, Belo Horizonte, Minas Gerais, Brazil; 5 Yale Department of Internal Medicine, Yale University School of Medicine, Yale University, New Haven, Connecticut, United States of America; Medical College of Wisconsin, United States of America

## Abstract

**Background:**

The role of the immune response in influencing leptospirosis clinical outcomes is not yet well understood. We hypothesized that acute-phase serum cytokine responses may play a role in disease progression, risk for death, and severe pulmonary hemorrhage syndrome (SPHS).

**Methodology/Principal Findings:**

We performed a case-control study design to compare cytokine profiles in patients with mild and severe forms of leptospirosis. Among patients hospitalized with severe disease, we compared those with fatal and nonfatal outcomes. During active outpatient and hospital-based surveillance we prospectively enrolled 172 patients, 23 with mild disease (outpatient) and 149 with severe leptospirosis (hospitalized). Circulating concentrations of pro- and anti-inflammatory cytokines at the time of patient presentation were measured using a multiplex bead array assay. Concentrations of IL-1β, IL-2, IL-4, IL-6, IL-8, IL-10, IL-17A, and TNF-α were significantly higher (*P*<0.05) in severe disease compared to mild disease. Among severe patients, levels of IL-6 (*P*<0.001), IL-8 (*P* = 0.0049) and IL-10 (*P*<0.001), were higher in fatal compared to non-fatal cases. High levels of IL-6 and IL-10 were independently associated (*P*<0.05) with case fatality after adjustment for age and days of symptoms. IL-6 levels were higher (*P* = 0.0519) among fatal cases who developed SPHS than among who did not.

**Conclusion/Significance:**

This study shows that severe cases of leptospirosis are differentiated from mild disease by a “cytokine storm” process, and that IL-6 and IL-10 may play an immunopathogenic role in the development of life-threatening outcomes in human leptospirosis.

## Author Summary

Leptospirosis is a tropical bacterial disease that is transmitted to humans from infected animals. Leptospirosis symptoms can range from mild fever to fatal disease forms, such as massive bleeding into the lungs, called Severe Pulmonary Hemorrhage Syndrome (SPHS). It is not known what determines the severity of leptospirosis, but we hypothesized that it may be influenced by differences in the type and concentration of signaling proteins called cytokines that are produced by the immune system in response to infection. We collected blood from patients with mild and severe leptospirosis, and compared the concentration of eight different cytokines circulating in the blood. We found that patients with severe leptospirosis had higher levels of most cytokines. Among patients who had severe forms, higher levels of specific cytokines called IL-6 and IL-8 were predictive of death even after statistical adjustment for age and number of days of symptoms prior to hospitalization. IL-6 was higher in patients who died from SPHS compared to those who died of other leptospirosis complications. This knowledge suggests that severe forms of leptospirosis may be due to a specific kind of immune response, which may lead to targeted therapies to reduce the impact of this disease.

## Introduction

Leptospirosis is a widely distributed zoonotic disease. Each year, approximately 500,000 cases of leptospirosis are reported worldwide [1], and as a consequence of predicted shifts in demographics and climate worldwide, the number of cases in urban areas is expected to increase [2]. The clinical manifestations of leptospirosis range from a mild acute febrile illness to severe or fatal forms, including Weil's syndrome (characterized by acute renal insufficiency, jaundice, and hemorrhage), which has a case fatality ratio of approximately 15% [1], [3]–[5]. A minority of patients develops a severe pulmonary hemorrhage syndrome (SPHS), which increases the risk of death to above 50% [6]. The pathology resulting from infection with *L. interrogans* is caused predominantly by activation of the innate immune response to disseminated bacteria, compromising lung and renal organs [3], [7]. It has been proposed that the variation in severity of cases may be due to the presence of virulence factors in certain serovars or strains of pathogenic *Leptospira* [8], differences in inoculum size that modify infecting pathogen burden [9] or differences in host immune response [10], [11] such as the pattern of cytokine production early in the course of the disease. Few studies have evaluated the immune response of patients with leptospirosis in order to obtain insights into the immunopathogenesis of disease progression. Previous investigations of the role of serum levels of cytokines and leptospirosis outcomes have shown conflicting results. A recent report showed that *L. interrogans* hemolysins induce IL-1β, IL-6 and TNF-α proinflammatory serum cytokine production in human and murine macrophages [12], and a study of whole human blood showed that virulent *Leptospira* were potent inducers of TNF-α and IL-6 through a Toll-like receptor-dependent mechanism [13]. High levels of TNF-α were also shown in patients hospitalized with leptospirosis [14], and an association was found between high levels of circulating TNF-α and an increased risk of lung involvement, bleeding and death [15], but a follow-up study by this group suggested that a high ratio of TNF-α to IL-10 may be a marker of lower severity [16]. Likewise, Kyriakidis et al. demonstrated an association between higher TNF-α levels and pulmonary hemorrhage [17]. However, the same investigation found that levels of TNF-α were significantly lower in fatal cases compared to nonfatal cases, while IL-10 levels were significantly higher among fatal cases; as a result, the authors proposed the use of a high ratio of IL-10 to TNF-α as a marker for leptospirosis severity.

These divergent findings may result from the use of different laboratory approaches to measure cytokine concentrations, and the small numbers of patients studied. In addition, none of the previous studies used multivariable analysis to understand independent associations and none included non-hospitalized patients with mild leptospirosis. A more detailed examination of the cytokine profile in well-characterized patients with disease of different severity is needed in order to better understand the role of cytokines in the immunopathogenesis of leptospirosis. In this study we evaluated a large panel of cytokines to describe the immune response of patients hospitalized with leptospirosis in comparison to ambulatory patients with mild disease.

## Methods

### Ethics statement

The study was approved by the Committee on Ethics in Research of the Oswaldo Cruz Foundation of Salvador, Bahia, the Brazilian National Committee on Ethics in Research, Ethics review committees of Hospital Couto Maia and Yale University. Written informed consent was obtained from all participants.

### Patients and study design

Between July 2006 and July 2010, we performed active hospital-based surveillance for severe leptospirosis in the state reference hospital for infectious diseases in Salvador, Brazil and identified 379 patients admitted with laboratory-confirmed leptospirosis. Of these, 149 (39%) had availability of sera collected and stored at −70°C within 24 hours of admission, and were included in this study. Availability of this serum depended on whether a patient was admitted during the workweek. Between January 2009 and February 2011, we also performed active outpatient surveillance for acute febrile illness in an urgent care health center serving a slum community in Salvador, Brazil. We identified 23 patients with laboratory-confirmed leptospirosis who had a self-limiting illness and did not require hospitalization (“mild disease”), all of whom had sera collected and stored at −70°C on the day of outpatient medical care.

Clinical data related to disease presentation and clinical outcome were extracted by review of patient records using a standardized questionnaire and entered into EpiInfo. The primary outcome of interest was death from any cause during hospitalization. The secondary outcome was SPHS, defined as chart documentation or direct observation of massive hemoptysis (≥approximately 250 cc in a single episode).

### Laboratory confirmation

Laboratory confirmation of severe disease was performed using a microscopic agglutination test (MAT), ELISA (Bio-Manguinhos, Rio de Janeiro, Brazil), or blood culture. The MAT panel included 10 reference strains and a local isolate, *Leptospira interrogans* serovar Copenhageni strain Fiocruz L1–130, representing nine serovars and nine serogroups. This panel effectively identified most locally circulating *Leptospira*, 90% of which are *L. interrogans* serovar Copenhageni [18]. Mild disease was confirmed using MAT and ELISA. To ensure specificity of the diagnosis of mild leptospirosis, we used an MAT battery of 26 strains (23 serogroups and 25 serovars). MAT confirmation criteria included seroconversion or a fourfold rise in titers between acute and convalescent sera obtained on the day of admission and after 14–30 days of convalescence, or a titer of ≥1∶800 in one or more samples [18], [19].

### Flow cytometer multiplex cytokine assays

Cytokines in sera from the leptospirosis patients were analyzed using the Human Th1/Th2 (IL-2, IL-4, IL-5, IL-10, TNF-α and IFN-γ), and inflammatory Cytometric Bead Array (CBA) Cytokine Kits (TNF-α, IL-1β, IL-6, IL-8 and IL-12p70) and the Th17 Human CBA Flex Set (IL-17A) (all from BD Biosciences, San Jose, CA), following the manufacturer's instructions.

All samples were stored at −70°C in aliquots, thawed once, were tested in batches using a uniform panel of control sera in each assay to reduce variation. Data were acquired on a BD™ FACSCalibur flow cytometer (BD Biosciences) and analyzed with CellQuest software and the data were formatted using BD CBA software, with results based on a standard concentration curve.

### Statistical analysis

Clinical characteristics and cytokine concentrations were compared between patients with severe and mild disease using Wilcoxon rank sum, Chi square, or Fisher exact test, as appropriate. Among patients who were hospitalized with severe disease, comparisons of cytokine concentrations between severe nonfatal and severe fatal cases were first performed using univariate logistic regression of log-transformed cytokine concentrations, and expressed as an odds ratio (OR). Variables significant to P≤0.1, and non-significant variables for clinical characteristics which may influence patient outcomes such as age, gender, and use of antibiotics, were entered into a backward stepwise selection multivariable logistic regression model. Number of days of symptoms prior to admission was forced into the multivariable model as it is an important source of potential confounding when studying the relationship between cytokine concentrations and severity of outcomes. This approach was repeated in the subset of patients with fatal outcomes, to compare the cytokine profile of patients who died with SPHS and those who died from other leptospirosis complications. P values<0.05 were considered to indicate statistical significance. Analyses were performed using SAS software, version 9.2 (SAS Institute, Cary, NC). Data for all variables were available for all patients.

## Results

### Patient characteristics

Patient characteristics are described in Table 1. Patients who were hospitalized with severe disease were older and a higher percentage was male. These patients were also more delayed to present for care, with a median of 6 days of symptoms (IQR 4–7), compared to 2 days (IQR 1–4) for patients with mild disease. Among 149 severe leptospirosis patients, 124 (83%) survived and 25 (17%) had fatal outcomes. On initial presentation, patients with fatal disease had higher frequency of oliguria (68% vs. 35%, *P* = 0.0035), and had more severe anemia (median HCT 18.9 vs. 34.5, *P*<0.001) and thrombocytopenia (median platelets 45,000/µL vs. 85,000, *P* = 0.0130) when compared to hospitalized patients with nonfatal disease. Compared to hospitalized patients with nonfatal outcomes, patients with fatal disease had more frequent clinical outcomes of bleeding (72% vs. 38%, *P*<0.001), severe pulmonary hemorrhage syndrome (SPHS) (44% vs. 2%, *P*<0.001), and admission to the Intensive Care Unit (68% vs. 13%, *P*<0.001). In addition, fatal cases had higher maximum serum creatinine (median 5.6 mg/dL vs. 3.7, *P*<0.001), lower minimum platelet count (34,000/µL vs. 64,500, *P*<0.001), and lower minimum hematocrit (18.9 vs. 30.0, *P*<0.001). Among those with fatal outcomes, 11 patients (44%) died with SPHS and 14 (56%) died from other complications of leptospirosis.

**Table 1:** Characteristics of included patients.

Variable	Mild disease (N = 23)	Severe nonfatal disease (N = 124)	Fatal disease (N = 25)
	N (%) or median (IQR)		
**Demographics**			
Age^a^ ^b^	30 (15–44)	33 (23–45)	43 (42–52)
Male gender^a^	14 (61)	108 (87)	21 (85)
**Clinical presentation**			
Days of symptoms prior to presentation^a^	2 (1–4)	6 (4–7)	6 (4–7)
Fever	23 (100)	121 (98)	24 (96)
Jaundice^a^	2 (9)	108 (87)	21 (84)
Oliguria^a^ ^b^	4 (17)	44 (35)	17 (68)
Serum creatinine (mg/dL)	NA	3.3 (1.7–5.7)	3.1 (2.2–6.0)
Total WBC (×1000/µL)	NA	13.5 (10.0–18.1)	13.3 (11.0–19.9)
Hematocrit^b^	NA	34.5 (31.0–38.0)	18.9 (14.7–27.2)
Platelets (×1000)^b^	NA	85 (51.1–139)	45 (31.1–63)
**Clinical course and outcomes**			
Severe pulmonary hemorrhage syndrome^a^ ^b^	0 (0)	3 (2)	11 (44)
Any bleeding^a^ ^b^ ^c^	1 (4)	47 (38)	18 (72)
Minimum platelet count (×1000/µL)^b^	NA	64.5 (40.0–103.0)	34.0 (20.0–45.0)
Minimum hematocrit (×1000/µL)^b^	NA	30.0 (24.6–35.1)	18.9 (14.7–27.2)
Maximum serum creatinine (mg/dL)	NA	3.7 (2.0–5.5)	5.6 (3.5–8.1)
Days of hospitalization	0 (NA)	8 (6–10)	5 (1.5–20)
Treatment with antibiotics	0 (NA)	96 (77)	18 (72)
Hemodialysis^b^	0 (NA)	25 (20)	12 (48)
Admission to Intensive Care Unit^b^	0 (NA)	17 (13)	17 (68)

N, number; IQR, inter-quartile range.

a *P*<0.05 comparing patients with mild disease against all hospitalized patients.

b *P*<0.05 comparing hospitalized patients with severe nonfatal disease against hospitalized patients with fatal disease.

c Any bleeding, including pulmonary or gastrointestinal hemorrhage, mild hemoptysis, epistaxis, or gingival bleeding.

### Cytokine concentrations and disease severity

**Figure 1:**
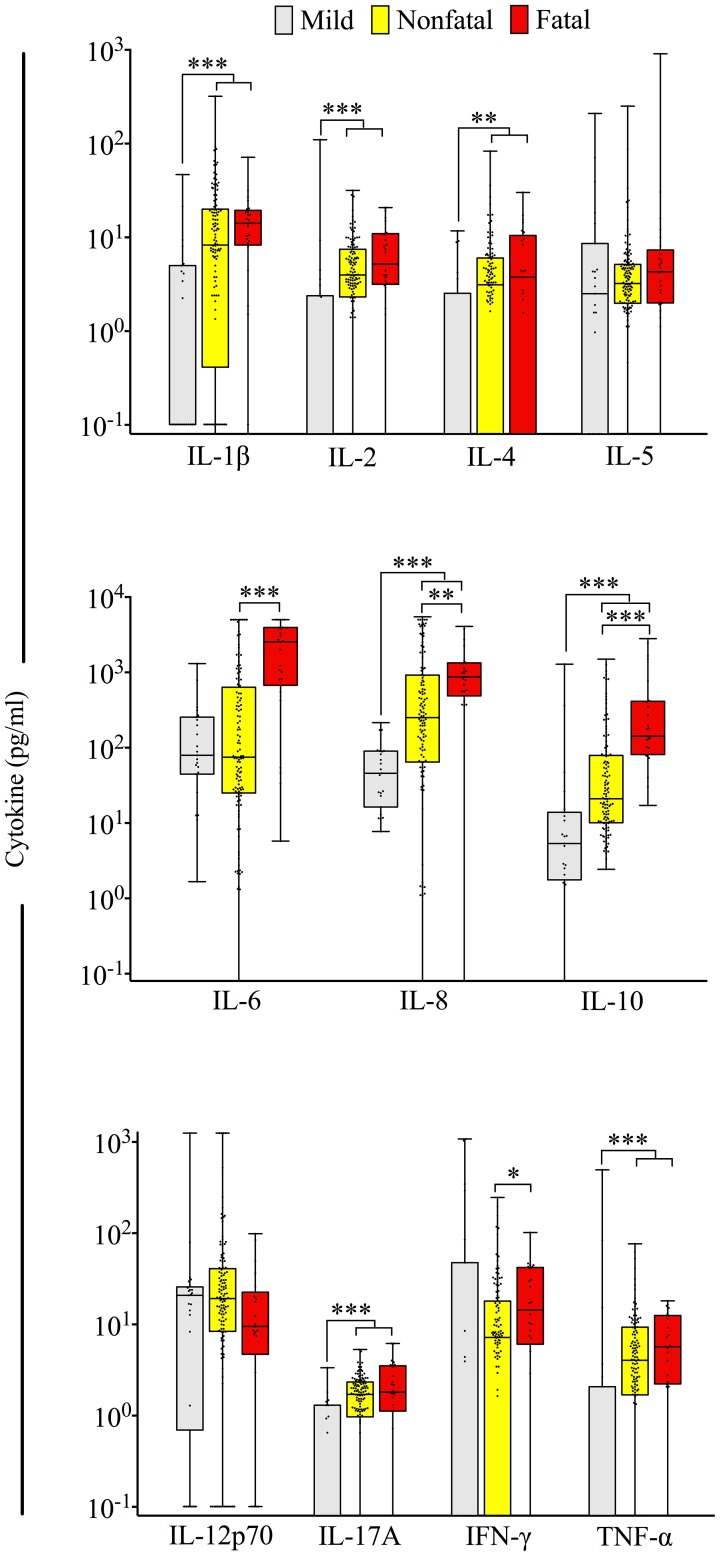
Comparison of serum cytokine concentrations in mild, nonfatal severe, and fatal leptospirosis. Box plots of serum cytokine concentrations among studied patients with mild, nonfatal severe, and fatal leptospirosis (gray, yellow, and red boxes, respectively). The bottom, median, and top lines of the box mark the 25th, 50th, and 75th percentiles, respectively. The vertical line with whiskers shows the range of values. Dots show individual data points. * *P*<0.05, ** *P*<0.01, *** *P*≤0.001.

There was a significant correlation between disease severity (mild, severe nonfatal, and fatal disease) and higher concentrations of a broad range of cytokines, including IL-1ß, IL-2, IL-4, IL-6, IL-8, IL-10, IL-17A, and TNF-α (Figure 1). Compared to patients with mild illness, hospitalized patients had higher concentrations of IL-1β (median 0.1 vs. 9.6 pg/mL, *P*<0.001), IL-2 (0.0 vs. 4.0 pg/mL, *P*<0.001), IL-4 (0.0 vs. 3.1 pg/mL, *P* = 0.0011), IL-8 (45.6 vs. 364.6 pg/mL, *P*<0.001), IL-10 (5.3 vs. 31.8 pg/mL, *P*<0.001), and TNF-α (0.0 vs. 4.1 pg/mL, *P*<0.001). Compared to hospitalized patients with nonfatal outcomes, patients with fatal outcomes had significantly higher concentrations of serum IL-6 (median 74.7 vs. 2536.6 pg/mL, *P*<0.001), IL-8 (251.1 vs. 873.9 pg/mL, *P* = 0.0049), IL-10 (21.0 vs. 142.0 pg/mL, *P*<0.001), and IFN-γ (7.2 vs. 14.4 pg/mL, *P* = 0.0195). There was no significant difference in the concentrations of TNF-α between fatal and nonfatal hospitalized patients (median 4.0 vs. 5.7 pg/mL, *P* = 0.6601), however the ratio of IL-10 to TNF-α was significantly higher in fatal cases compared to severe nonfatal cases (32.0 vs. 4.8, *P* = 0.0019).

These cytokine concentrations were entered into a stepwise selected multivariable logistic regression model to predict death among hospitalized patients with severe leptospirosis (Table 2). In adjusted analysis, higher concentrations of IL-6 (OR, 1.73; 95% confidence interval [CI], 1.09–2.73) and IL-10 (OR, 1.9; 95% CI, 1.16–3.11) were independently associated with death after adjusting for age and days of symptoms before hospitalization. Although duration of illness before hospitalization was not significantly associated with death, we kept this variable in the model because cytokine concentrations may be influenced in part by duration of illness prior to identification.

**Table 2:** Immunologic predictors of death among hospitalized patients.

	Odds ratio for death (95% CI)	
Factor	Univariable model	Multivariable model
**Clinical variables**		
Age	**1.047 (1.015–1.081)**	**1.057 (1.015–1.101)**
Male gender	0.778 (0.236–2.559)	–
Days of symptoms	0.984 (0.818–1.184)	1.274 (0.960–1.691)
Use of antibiotics	1.333 (0.506–3.515)	–
**Serum cytokines**		
IL-1ß	1.144 (0.878–1.492)	–
IL-2	1.509 (0.787–2.895)	–
IL-4	1.435 (0.738–2.791)	–
IL-5	1.330 (0.884–2.002)	–
**IL-6**	**1.781 (1.354–2.343)**	**1.726 (1.090–2.731)**
IL-8	**1.607 (1.153–2.239)**	–
**IL-10**	**2.088 (1.526–2.858)**	**1.895 (1.155–3.107)**
Il-12p70	0.871 (0.728–1.042)	–
Il-17A	2.505 (0.824–7.610)	–
IFN-γ	1.349 (0.845–2.153)	–
TNF-α	1.087 (0.638–1.853)	–

–, Not selected for entry into multivariable model. Bold font signifies significant association with death. Odds ratio is expressed per log increment of cytokine concentration.

### Cytokine concentration and severe pulmonary hemorrhage

**Figure 2:**
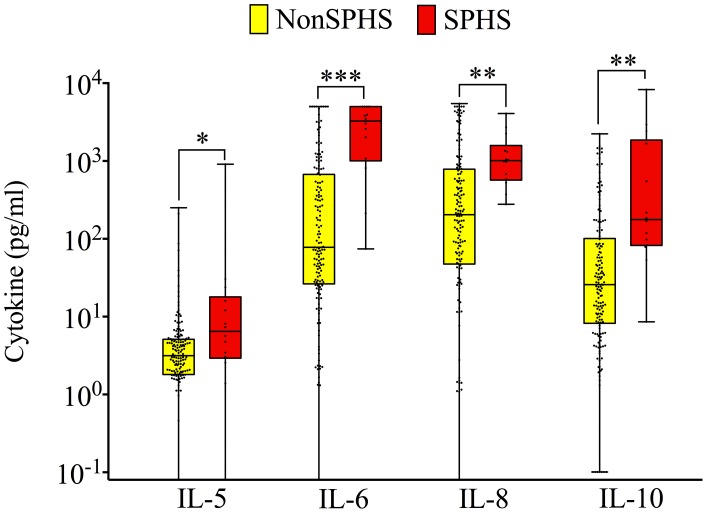
Comparison of serum cytokine concentrations in hospitalized patients with and without severe pulmonary hemorrhage syndrome. Box plots of selected serum cytokine concentrations among hospitalized patients with and without severe pulmonary hemorrhage syndrome (SPHS) (red and yellow boxes, respectively). The bottom, median, and top lines of the box mark the 25th, 50th, and 75th percentiles, respectively. The vertical line with whiskers shows the range of values. Dots show individual data points. * *P*<0.05, ** *P*<0.01, *** *P*≤0.001.

Among all hospitalized patients, those with SPHS had significantly higher levels of serum IL-5 (median 6.5 vs. 3.2 pg/mL, *P* = 0.0186), IL-6 (3262.9 vs. 77.2 pg/mL, *P*<0.001), IL-8 (1012.9 vs. 293.9 pg/mL, *P* = 0.0046), and IL-10 (163.0 vs. 26.8 pg/mL, *P* = 0.0019) (Figure 2). Only three patients with SPHS survived; therefore death and SPHS were highly collinear. Because of this collinearity, we restricted the evaluation of an association between cytokine levels and SPHS to the 25 patients who had fatal outcomes. In univariate analysis, fatalities from SPHS had higher levels of IL-6, IL-8, and IL-10 in comparison to fatalities from other leptospirosis-related complications (*P*<0.05). After adjusting for age and duration of symptoms, serum IL-6 remained associated with SPHS among patients with fatal outcomes (median 3796.9 pg/mL for death from SPHS patients vs. 906.7 pg/mL for those who died from other complications, *P* = 0.0519).

## Discussion

In this investigation, we describe a detailed examination of cytokine production in a large, well-characterized series of patients with laboratory-confirmed leptospirosis of differing severity. This is the first study to perform multivariable analysis to identify independent associations between specific cytokines and severe outcomes, allowing us to delineate the specific contribution of individual cytokines among the milieu of co-circulating cytokines. In addition, we adjusted for age and duration of illness at presentation because these factors, which may influence severity of disease, may also modify the circulating cytokine profile. Although cytokine response may also be influenced by differences in race [20], [21], we did not adjust for race, as in the state of Bahia, 77% of the population self-classify as black or mixed race (“parda”) [22], and racial distinctions are less well defined as in other contexts. Our study provides evidence that specific patterns of cytokine response are associated with different clinical outcomes, such as the need of hospitalization, and death, as well as with development of severe pulmonary hemorrhage syndrome.

We found that in patients with mild leptospirosis, there was some measurable elevation of pro-inflammatory cytokines, particularly IL-6 and IL-8. However, compared to mild leptospirosis, our results suggest that severe disease manifestations requiring hospitalization are distinguished by a broad activation of both pro- and anti-inflammatory cytokines. The pattern of generalized cytokine activation that we describe in patients with severe disease is consistent with a “cytokine storm” similar to that seen in other inflammatory conditions including bacterial sepsis [23], [24].

Although severe leptospirosis was associated with cytokine storm, we found that specific cytokine signatures were associated with the most severe outcomes. Among patients hospitalized with severe leptospirosis, increased concentrations of IL-6, IL-8, IL-10 and IFN-γ were associated with fatal outcomes in univariate analysis. After adjusting for age and duration of illness before hospitalization, IL-6 and IL-10 were both independent predictors of death. Thus, it is possible that the progression from a non-specific exacerbated immune response to a Th2-dominant adaptive immune response, which includes inhibition of the Th1 response by overproduction of IL-10, plays an important immunopathogenic role in determining the risk of death from leptospirosis. This observation is consistent with results observed in animal models of leptospirosis in which production of IL-10 was significantly associated with increased case fatality [25]. Furthermore, previous studies of patients with leptospirosis had also identified higher levels of IL-10 to be associated with death [17]. IL-10 is known to be an important regulator of inflammation in sepsis [26], and plays an important role in down-regulating the expression of monocyte-derived TNF-α and IL-1 [27]–[29]. IL-10 inhibits nuclear factor kappa B (NF-κB), the surface expression of major histocompatibility complex class II molecules, nitric oxide synthesis and down-regulation of TNF-α receptors [30] after LPS stimulation. Notably, we also found that a high ratio of IL-10 to TNF-α was associated with death from leptospirosis, driven predominantly by IL-10 levels. Fatal leptospirosis has been previously associated with both high [15] and low levels of TNF-α [17]. In the present study, we did not observe significantly higher levels of TNF-α in patients who died in comparison to those who survived; several patients in our study had undetectable concentrations of TNF-α. In addition, we did not measure soluble TNF receptor-1 (sTNFR1), which may have led to decreased detection of circulating TNF-α due to unmeasured bound TNF-α.

We found that higher levels of IL-5, IL-6, IL-8, and IL-10 were associated with SPHS in univariate analysis. After studying only fatal cases and adjusting for age and disease duration before presentation, we still found an association between higher IL-6 and SPHS, suggesting a potential specific role of IL-6 in the pathophysiology of this important clinical syndrome. A potential role for IL-6 in determining the severity of acute long injury (ALI) has been previously observed in other settings. Parsons et al. [31] observed that in patients with acute respiratory distress syndrome, increased levels of IL-6 and IL-8 at baseline were associated with increased risk of death. Ahuaja et al. [32] demonstrated that in a mouse model of ALI, IL-6 may play a direct pathophysiological role in increasing lung inflammation and poor outcomes. We cannot discard the possibility of an independent association between other Th2 cytokines and SPHS because we were underpowered to detect multiple significant associations in the multivariable analysis.

The design of our study does not allow us to determine if the pattern of cytokines observed in patients with severe outcomes reflects a mechanistic role as mediators of pathogenesis, or if they are only markers of disease severity or progression. A prospective study of the temporal evolution of the cytokine response to leptospirosis would be an appropriate next step to better understand to what extent cytokine production is a key component in the pathogenesis of severe leptospirosis, and may help identify targets for potential therapeutic intervention.

In summary, our findings suggest that severe leptospirosis induces a “cytokine storm” during the first days of infection, and that an immune response with overproduction of IL-10 and IL-6 cytokines may be implicated in the mechanism of severe leptospirosis forms including SPHS, an important complication with a high fatality ratio. These findings, if confirmed by further studies of the temporal evolution of cytokine response, may help guide new therapeutic approaches to reduce morbidity and mortality due to severe leptospirosis.

## Supporting Information

Checklist S1 STROBE checklist.
(DOC)
