# Supplementary material for: Cytokine Response Signatures in Disease Progression and Development of Severe Clinical Outcomes for Leptospirosis
Source: PLoS Negl Trop Dis. 2013 Sep 19;7(9):e2457. doi: 10.1371/journal.pntd.0002457 (PMC3777885; doi:10.1371/journal.pntd.0002457)
Supplement: Checklist S1 — STROBE checklist. (DOC) [file pntd.0002457.s001.doc]

PNTD-D-13-00705R1

STROBE checklist for a Case-control study

|  | Item No | Recommendation |
| --- | --- | --- |
| **Title and abstract** | 1 | (*a*) Indicate the study’s design with a commonly used term in the title or the abstract  The study uses a case control analysis, and this is described in the abstract. |
|  |  | (*b*) Provide in the abstract an informative and balanced summary of what was done and what was found  Done. |
| Introduction | | |
| Background/rationale | 2 | Explain the scientific background and rationale for the investigation being reported  Done. |
| Objectives | 3 | State specific objectives, including any prespecified hypotheses  Done. |
| Methods | | |
| Study design | 4 | Present key elements of study design early in the paper  Done. |
| Setting | 5 | Describe the setting, locations, and relevant dates, including periods of recruitment, exposure, follow-up, and data collection  Done. |
| Participants | 6 | (*a*) *Cohort study*—Give the eligibility criteria, and the sources and methods of selection of participants. Describe methods of follow-up  *Case-control study*—Give the eligibility criteria, and the sources and methods of case ascertainment and control selection. Give the rationale for the choice of cases and controls  *Cross-sectional study*—Give the eligibility criteria, and the sources and methods of selection of participants  Done. |
|  |  | (*b*) *Cohort study*—For matched studies, give matching criteria and number of exposed and unexposed  *Case-control study*—For matched studies, give matching criteria and the number of controls per case  This study was not a matched study. |
| Variables | 7 | Clearly define all outcomes, exposures, predictors, potential confounders, and effect modifiers. Give diagnostic criteria, if applicable.  Definitions are included “Patients and study design” section of the Methods. |
| Data sources/ measurement | 8* | For each variable of interest, give sources of data and details of methods of assessment (measurement). Describe comparability of assessment methods if there is more than one group  Done. This is provided in the text of the Methods section. |
| Bias | 9 | Describe any efforts to address potential sources of bias  Done. |
| Study size | 10 | Explain how the study size was arrived at  This study used a sample of patients recruited using population-based surveillance for hospitalized and ambulatory patients with leptospirosis, over a period of 4 years. It is an exploratory study that uses as many patients as met the inclusion criteria within that surveillance period, and thus was not designed with an apriori sample size. |
| Quantitative variables | 11 | Explain how quantitative variables were handled in the analyses. If applicable, describe which groupings were chosen and why  Done. |
| Statistical methods | 12 | (*a*) Describe all statistical methods, including those used to control for confounding  Done. |
|  |  | (*b*) Describe any methods used to examine subgroups and interactions  Done. |
|  |  | (*c*) Explain how missing data were addressed  Done. – There were no missing data. |
|  |  | (*d*) *Cohort study*—If applicable, explain how loss to follow-up was addressed  *Case-control study*—If applicable, explain how matching of cases and controls was addressed  *Cross-sectional study*—If applicable, describe analytical methods taking account of sampling strategy  No matching was performed. |
|  |  | (*e*) Describe any sensitivity analyses  Does not apply. |

Continued on next page

| Results | | |
| --- | --- | --- |
| Participants | 13* | (a) Report numbers of individuals at each stage of study—eg numbers potentially eligible, examined for eligibility, confirmed eligible, included in the study, completing follow-up, and analysed  Done. |
|  |  | (b) Give reasons for non-participation at each stage  Done. |
|  |  | (c) Consider use of a flow diagram  This was considered, but the study size and design were considered sufficiently small and simple that this would not add to the clarity of the work. |
| Descriptive data | 14* | (a) Give characteristics of study participants (eg demographic, clinical, social) and information on exposures and potential confounders  Done. |
|  |  | (b) Indicate number of participants with missing data for each variable of interest  No missing data |
|  |  | (c) *Cohort study*—Summarise follow-up time (eg, average and total amount)  Does not apply |
| Outcome data | 15* | *Cohort study*—Report numbers of outcome events or summary measures over time  Does not apply |
|  |  | *Case-control study—*Report numbers in each exposure category, or summary measures of exposure  Done. |
|  |  | *Cross-sectional study—*Report numbers of outcome events or summary measures  Does not apply. |
| Main results | 16 | (*a*) Give unadjusted estimates and, if applicable, confounder-adjusted estimates and their precision (eg, 95% confidence interval). Make clear which confounders were adjusted for and why they were included  Unadjusted estimates provided, but the IQR was used instead of 95% CI). |
|  |  | (*b*) Report category boundaries when continuous variables were categorized  Does not apply. |
|  |  | (*c*) If relevant, consider translating estimates of relative risk into absolute risk for a meaningful time period  Does not apply. |
| Other analyses | 17 | Report other analyses done—eg analyses of subgroups and interactions, and sensitivity analyses  Does not apply. |
| Discussion | | |
| Key results | 18 | Summarise key results with reference to study objectives  Done. |
| Limitations | 19 | Discuss limitations of the study, taking into account sources of potential bias or imprecision. Discuss both direction and magnitude of any potential bias  Done. |
| Interpretation | 20 | Give a cautious overall interpretation of results considering objectives, limitations, multiplicity of analyses, results from similar studies, and other relevant evidence  Done. |
| Generalisability | 21 | Discuss the generalisability (external validity) of the study results  Done. |
| Other information | | |
| Funding | 22 | Give the source of funding and the role of the funders for the present study and, if applicable, for the original study on which the present article is based  Done. |

*Give information separately for cases and controls in case-control studies and, if applicable, for exposed and unexposed groups in cohort and cross-sectional studies.

**Note:** An Explanation and Elaboration article discusses each checklist item and gives methodological background and published examples of transparent reporting. The STROBE checklist is best used in conjunction with this article (freely available on the Web sites of PLoS Medicine at http://www.plosmedicine.org/, Annals of Internal Medicine at http://www.annals.org/, and Epidemiology at http://www.epidem.com/). Information on the STROBE Initiative is available at www.strobe-statement.org.
